# Majoramputationen der unteren Extremität

**DOI:** 10.1007/s00113-024-01530-1

**Published:** 2025-02-04

**Authors:** Patrick Schröter, Marc Hückstädt, Steffen Langwald, Bianca Schröter, Philipp Kobbe

**Affiliations:** Klinik für Unfall- und Wiederherstellungschirurgie, BG Klinikum Bergmannstrost Halle, Merseburger Straße 165, 06112 Halle, Deutschland

**Keywords:** Oberschenkel, Unterschenkel, Exartikulation, Amputationsstumpf, Plastische Chirurgie, Thigh, Lower leg, Disarticulation, Amputation stumps, Plastic surgery

## Abstract

In Deutschland gibt es jährlich etwa 18.500 Majoramputationen an der unteren Extremität, meist transtibial oder transfemoral. Für eine erfolgreiche Rehabilitation sind Kenntnisse über die angestrebte optimale Stumpfbeschaffenheit und spezielle Amputationstechniken wichtig. Biomechanische Überlegungen bezüglich des Amputationsstumpfes spielen bei der auszuwählenden Operationstechnik ebenso eine entscheidende Rolle wie die Amputationsursache. Techniken wie die Myodese, Myoplastie oder die Gottschalk-Plastik helfen dabei, die Muskelspannung zu erhalten und die Prothesenkontrolle zu optimieren.

## Abstract (en)

In Germany there are approximately 18,500 major amputations of the lower extremity each year, primarily transtibial or transfemoral. For successful rehabilitation, knowledge about the desired optimal stump condition and specific amputation techniques are essential. Biomechanical considerations regarding the amputation stump play a crucial role in the selection of the surgical technique as do the reasons for the amputation. Techniques such as myodesis, myoplasty or Gottschalk plastic surgery help to maintain muscle tension and optimize prosthesis control.

## Lernziele

Nach Lektüre dieses Beitrags ...

- kennen Sie die häufigsten Ursachen für Majoramputationen der unteren Extremität.
- haben Sie die wichtigsten biomechanischen Aspekte der Ober- und Unterschenkelamputation kennengelernt.
- kennen Sie die häufigsten Amputationstechniken bei Ober- und Unterschenkelamputationen.
- kennen Sie die idealen Stumpfbeschaffenheiten zur Prothesenschaftnutzung.

## Einleitung

Amputationen oberhalb des Sprunggelenks werden als **Majoramputationen**

Majoramputationen

bezeichnet. Hauptursachen, in 73 % der Fälle, sind komplizierte Verläufe von **Diabetes mellitus**

Diabetes mellitus

und **arteriellen Verschlusskrankheiten**

arteriellen Verschlusskrankheiten.

Seltenere Gründe stellen chronische Osteitiden, Verletzungen oder Tumoren dar [1]. In Deutschland werden jährlich etwa 18.500 Majoramputationen durchgeführt. **Transtibiale Amputationen**

Transtibiale Amputationen

und **transfemorale Amputationen**

transfemorale Amputationen

sind die häufigsten Majoramputationen der unteren Extremitäten [2].

Für eine erfolgreiche Rehabilitation nach einer Majoramputation mit guter Prothesennutzung sind Kenntnisse über die optimale **Stumpfbeschaffenheit**

Stumpfbeschaffenheit

und verschiedene Amputationstechniken erforderlich. Es ist wichtig, die Ursachen der nötigen Amputation zu berücksichtigen. Hochgradig durchblutungsgestörte oder diabetesgeschädigte Extremitäten erfordern ein anderes Vorgehen als traumatisch geschädigte, ansonsten gesunde Extremitäten, um eine sichere Wundheilung und einen belastbaren Stumpf zu gewährleisten.

Schwere **Weichteilverletzungen**

Weichteilverletzungen

führen selten innerhalb von 24 h zur Amputationsnotwendigkeit, da moderne chirurgische Rekonstruktionsverfahren technisch nahezu immer einen anfänglichen Erhalt der Extremität gestatten. Sollten allerdings subtotale oder totale Amputationen aufgrund der Schwere der Verletzung eine chirurgische Amputation erzwingen, spricht man von einer primären traumatischen Amputation. Score-Systeme wie der Mangled Extremity Severity Score (MESS) und der Limb Salvage Index (LSI) bieten Unterstützung bei der Entscheidung über die Notwendigkeit einer Amputation [3]. Hierbei finden nicht nur die Verletzungen an der Extremität Berücksichtigung, sondern auch das Patientenalter und der durch das Trauma bedingte Schock [4, 5]. Die Anwendung der Score-Systeme erbringt im klinischen Alltag allerdings keine Vorteile, da eine prognostische Abschätzung der Funktionalität nach Extremitätenerhalt bzw. eine spätere Amputationsnotwendigkeit nicht hinreichend abgeschätzt wird [3]. Insbesondere **Nervenschäden**

Nervenschäden

und **Gefäßschäden**

Gefäßschäden

sind im individuellen Fall von entscheidender Bedeutung für den Verlauf. Die letztendliche Entscheidung für oder gegen die Amputation muss anhand klinischer Erfahrungen gestellt werden [6, 7].

**Sekundäre Amputationen**

Sekundäre Amputationen

erfolgen innerhalb von Tagen bis wenigen Wochen aufgrund großer Weichteildefekte, Infektionen oder Gefäß-Nerven-Verletzungen. Beim Erfordernis notwendiger Amputationen aufgrund eingetretener Komplikationen im Behandlungsverlauf, werden diese ab der 6. Woche nach dem Verletzungsereignis als **tertiäre Amputationen**

tertiäre Amputationen

bezeichnet. **Quartäre Amputationen**

Quartäre Amputationen

werden nach Monaten oder Jahren aufgrund von Osteomyelitis, Funktionseinbußen oder chronischen Schmerzen notwendig [8].

## Oberschenkelamputation

Transfemorale Amputationen durch die Diaphyse werden als Oberschenkelamputationen bezeichnet und sind indiziert, wenn eine Knieexartikulation oder eine transkondyläre Amputation nicht möglich ist. Aufgrund besserer Behandlungsmöglichkeiten der Amputationsursachen ist von 2005–2015 die Oberschenkelamputationsrate von 26,8 % auf 17,3 % der Majoramputationen gefallen [1].

Der knöcherne Stumpf wird mit einem myokutanen Weichteillappen bedeckt. Bei der **Myodese**

Myodese,

auch als **Myopexie**

Myopexie

bezeichnet, werden die Muskeln direkt an den Knochen fixiert, meist durch transossäre Nähte oder kräftige Periostnähte. Bei der Myoplastie werden die Muskeln miteinander über dem knöchernen Stumpfende vernäht. Die Kombination aus beiden Techniken gewährleistet eine stabile und gut gepolsterte distale Stumpfregion. Wichtig ist es, die Muskelspannung aufrechtzuerhalten, was die **Prothesenkontrolle**

Prothesenkontrolle

und -nutzung optimiert sowie das Risiko von **Muskelatrophie**

Muskelatrophie

verringert. Durch die Myopexie, das chirurgische Fixieren der Muskulatur am Knochen, wird zudem eine Dislokation bei Eigenbewegung der Muskulatur verhindert.

Die optimale **Weichteildeckung**

Weichteildeckung

des knöchernen Stumpfendes zeichnet sich durch geringe Weichteilverschieblichkeit und ein angemessenes Volumen des Weichteilmantels aus. Zu viel **Weichteilmasse**

Weichteilmasse

distal des knöchernen Stumpfes resultiert in einer ungünstigen Führung des Stumpfes durch den Prothesenschaft [9].

### Biomechanik des Oberschenkelstumpfes

Biomechanische Untersuchungen zeigen, dass ein längerer Reststumpf von erheblichem Vorteil ist. Je kürzer der knöcherne Stumpf, desto geringer der **funktionelle Hebelarm**

funktionelle Hebelarm

und desto höher der nötige Kraftaufwand. Abhängig von der **Stumpflänge**

Stumpflänge

benötigt ein Oberschenkelamputierter bis zu 100 % mehr Energie, um gehen zu können. Zudem überwiegen die Muskeln für die Außenrotation und Hüftbeugung bei kurzen Stümpfen. Dies führt zu einer zunehmenden Lateralisierung, Außenrotation und Hüftbeugefehlstellung des knöchernen Stumpfes [10]. Die resultierenden Fehlstellungen des knöchernen Stumpfes können im **Prothesenschaft**

Prothesenschaft

erhebliche mechanische Beeinträchtigungen bewirken. Selbst bei optimaler Muskel-Knochen-Fixierung finden im Rahmen der Schrittabwicklung Relativbewegungen des Femurstumpfes zum Prothesenschaft innerhalb des Stumpfweichteiles statt. Ultraschalluntersuchungen in Schaftsystemen haben einen Positionswechsel des Femurstumpfendes bis zu 12°, bezogen auf das Hüftgelenk, bei Lastübernahme nachgewiesen. Bei einer knöchernen Stumpflänge von 25 cm bewegt sich das Femurende bis zu 5 cm innerhalb des Weichteilmantels [11].

Aus diesem Grund weisen längere knöcherne Stümpfe verbesserte Gehparameter durch effizientere** Kraftübertragung**

Kraftübertragung

und bessere **Balance**

Balance

auf. Zudem besteht ein niedrigeres Risiko für eine **Hüftbeugekontraktur**

**Abb. 1:**
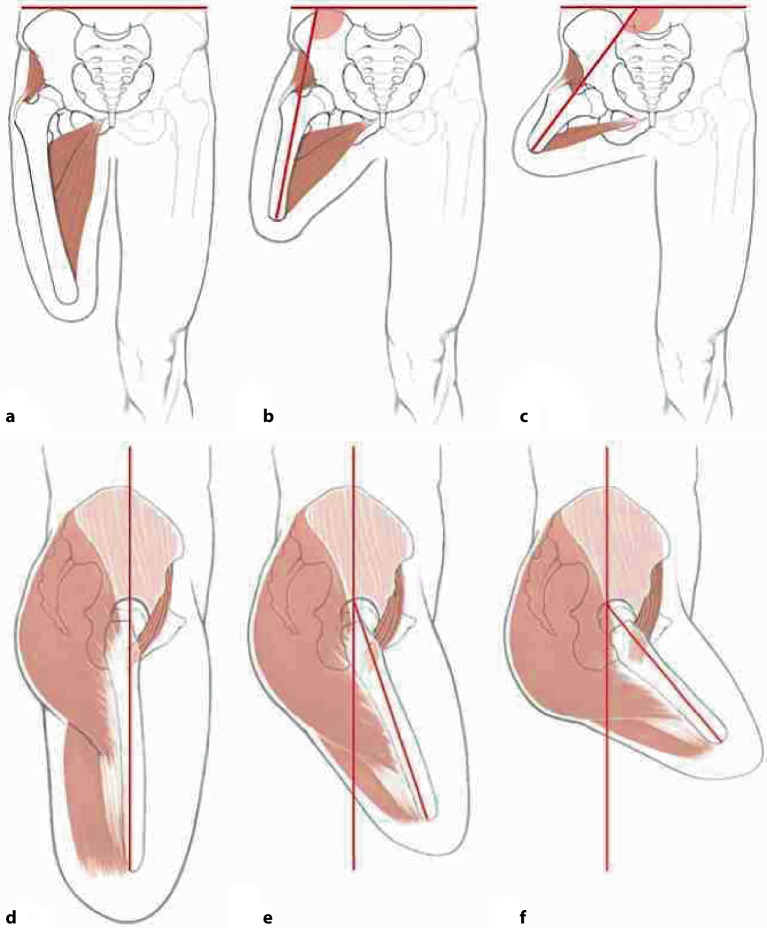
Bei zunehmend kürzerem Oberschenkelstumpf überwiegt das Muskelgleichgewicht zugunsten der Hüftbeuger (**a**–**c**). Das Überwiegen des M. iliopsoas führt zu einer Hüftbeugefehlstellung. In der frontalen Ebene zeigt sich bei kürzeren Oberschenkelstümpfen aufgrund des zunehmenden Verlustes der Adduktorenmuskulatur eine Abduktionsfehlstellung. Zudem führt die Kombination der Zugrichtung der Glutäusmuskulatur und des M. iliopsoas zu einer Außenrotationsfehlstellung, wie sie auch bei subtrochantären Femurfrakturen bekannt ist. (Aus Baumgartner [13])

Hüftbeugekontraktur,

und der lange Hebelarm mit den verbliebenen Restmuskeln zeigt eine deutlich physiologischere räumliche Position des Femurstumpfes bei der Prothesennutzung auf (Abb. 1 und 2; [12]).

**Abb. 2:**
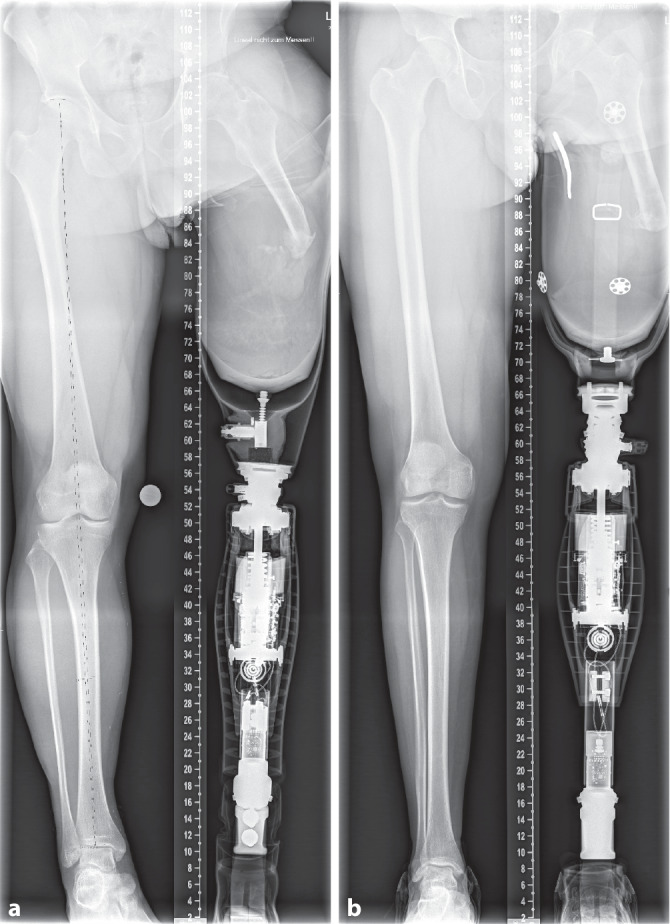
Diese röntgenologischen Ganzbeinaufnahmen mit axialer Vollbelastung im Prothesenschaft zeigen typische Femurabweichungen mit Abduktions‑, Außenrotations- und Anteversionsfehlstellung. Zudem sind bei beiden Patienten die kaudalen Stumpfweichteile deutlich überhängend und erschweren eine Führung des Stumpfes im Schaft

#### Merke

Je kürzer der Femurstumpf, desto größer ist die resultierende Abduktions‑, Außenrotations- und Hüftbeugefehlstellung.

Die Operationstechniken der Muskelfixierung (Myoplastie, Myopexie und die Refixierung des M. adductor magnus nach der **Gottschalk-Technik**

Gottschalk-Technik

am distalen lateralen Oberschenkelstumpf [14]) sind notwendig, um diesen Fehlstellungen entgegenwirken zu können.

Der M. adductor magnus stabilisiert den Oberschenkel in seiner anatomischen Position. Der Verlust des distalen Drittels resultiert in einem 70 %igen Kraftverlust des effektiven Hebelarms und bedingt die **Abduktionsfehlstellung**

**Abb. 3:**
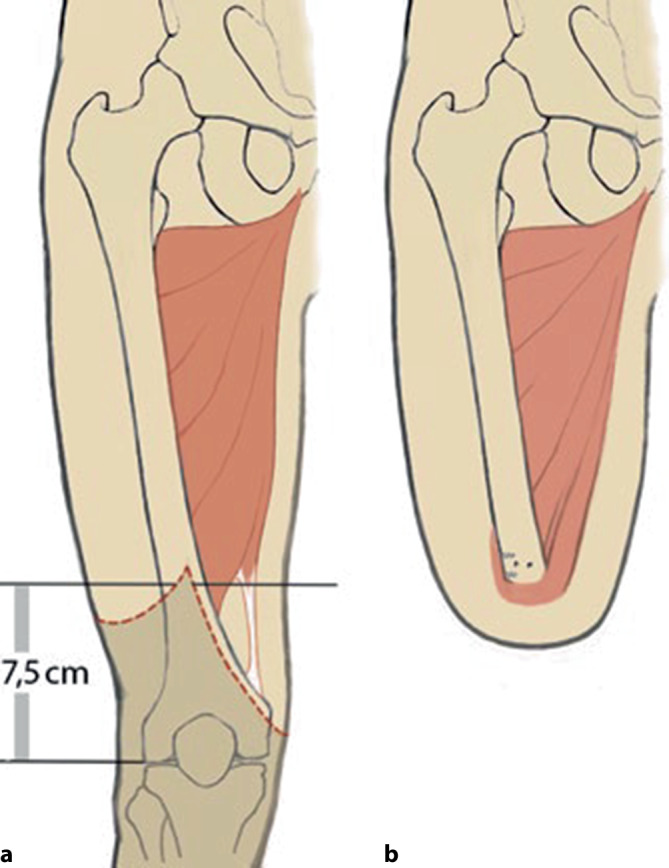
Der M. adductor magnus wird über das distale Stumpfende des Femurs geführt und unter maximaler Adduktion im Hüftgelenk lateral periostal und transossär refixiert. Dadurch kann eine physiologische Stellung des Oberschenkelknochens im Stumpf erreicht werden. Idealerweise wird bei dieser Technik im Rahmen der Amputation der Sehnenspiegel des M. adductor magnus vom Tuberculum adductorium gelöst und am distalen Femur fixiert. Diese Operationstechnik ist nur bei langen Oberschenkelstümpfen möglich. *Gestrichelt* ist die Stumpflänge dargestellt; die Schnittführung der asymmetrischen Hautlappen ist *farblich markiert*. – Aus [13]

Abduktionsfehlstellung

des Femurs bei transfemoralen Amputationen. Eine muskelschonende transfemorale Amputation, die den M. adductor magnus intakt lässt, verhindert diese Abduktion. Bei der Durchführung der Gottschalk-Plastik wird das Femur in die maximal mögliche Adduktion gebracht und die Adduktorenmuskulatur am lateralen distalen Stumpfende auf einer Länge von etwa 6–8 cm fixiert (Abb. 3; [14]).

### Knieexartikulation und transepiphysäre distale Femuramputation

Die biomechanischen Probleme, die bei einer transfemoralen diaphysären Oberschenkelamputation auftreten und u. a. von Gottschalk beschrieben wurden, sind bei einer Knieexartikulation oder einer transepiphysären Amputation nicht vorhanden. Dies liegt v. a. daran, dass die **Adduktorenmuskulatur**

**Abb. 4:**
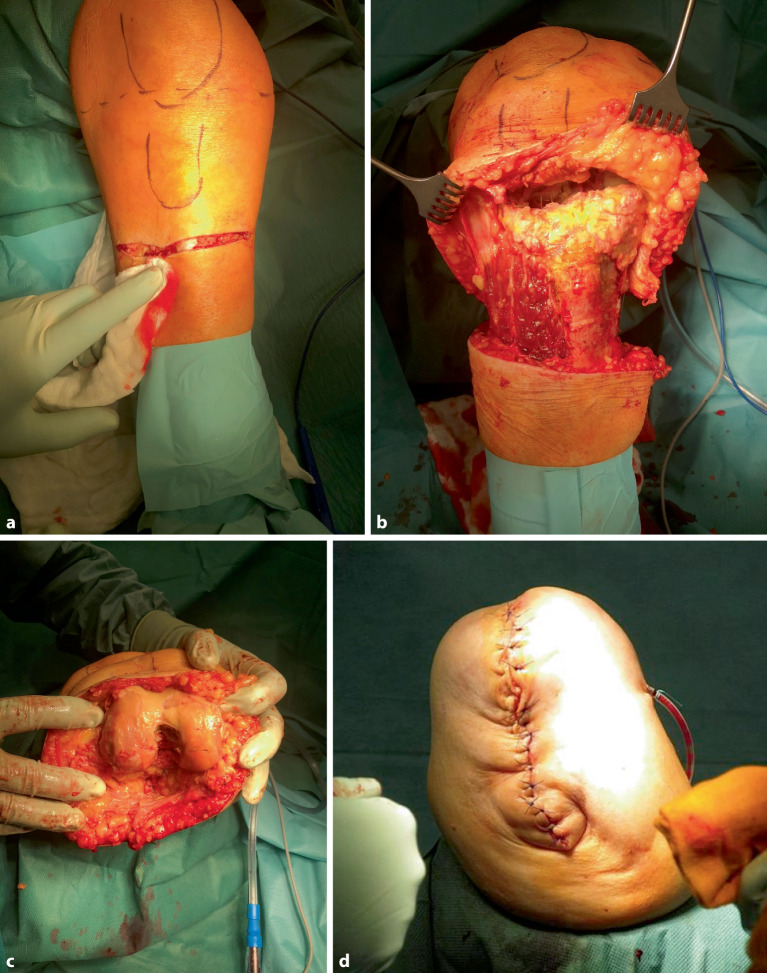
Bei der Knieexartikulation erfolgt das zirkuläre Durchtrennen der Haut- und Unterhaut ca. 12 cm unterhalb der Kniegelenklinie (**a**). Anschließend wird subfaszial oder unmittelbar epiperiostal bis in den Kniegelenkspalt präpariert und der Unterschenkel durch das Durchtrennen aller Strukturen auf Kniegelekspalthöhe abgesetzt (**b**). Es werden alle Kniegelenkbinnenstrukturen, einschließlich der Kreuzbänder, reseziert (**c**). Der Hautschlauch wird nach kaudal-dorsal gezogen, und der längs verlaufende Wundverschluss legt die zukünftige Narbe nach interkondylär (**d**). Damit befindet sich die Narbe bei Prothesennutzung nicht in der Belastungszone

Adduktorenmuskulatur

vollständig mit ihrem Ansatz am Femur erhalten bleibt (Abb. 4). Dies führt zu einer nahezu anatomischen Position des Amputationsstumpfes im Raum [10, 14]. Zudem bietet die transepiphysäre Amputation durch die größere Auflagefläche eine deutlich bessere Belastbarkeit des Stumpfes im Vergleich zur diaphysären Amputation. Bei einer Knieexartikulation ist die Belastbarkeit des Stumpfes sogar vollständig gewährleistet, sofern die Weichteile unauffällig sind, da das Kondylenmassiv anatomisch intakt bleibt und die volle Körperlast tragen kann [10].

### Stumpfvoraussetzungen zur Prothesenversorgung

Beschrieben werden die idealen Stumpfbeschaffenheiten, an denen sich bei einer Amputationsstumpfbeurteilung orientiert werden soll. Das Ziel der Oberschenkelamputation ist ein schmerzfreier, infektionsfreier und belastbarer Stumpf. Die Beweglichkeit im Hüftgelenk soll uneingeschränkt sein und keine Hüftbeugekontraktur aufweisen [15].

Haut- und Unterhaut:

- gute Durchblutung (Rekapillarisierungszeit ca. 2–3 s),
- keine Sensibilitätsstörung der Haut,
- schmerzfrei,
- Haut/Narbe ist über dem Untergrund verschieblich und reizlos,
- Lastaufnahme wird vertragen.

Weichteile:

- fixierte Muskeldeckung des Stumpfes ohne zu viel Weichteilüberhang,
- die antagonisierende Muskulatur ist mit sich und dem Knochenende fixiert,
- große Gefäße sind kurz proximal des knöchernen Stumpfes chirurgisch abgesetzt,
- die Nerven (N. femoralis, N. saphenus, N. ischiadicus) sind hinreichend gekürzt und in die Weichteile verlagert.

Knöcherner Stumpf:

- die Femurresektionsfläche ist an den Kanten abgerundet, horizontal ausgerichtet und weist keine Osteophyten auf.

#### Merke

**Abb. 5:**
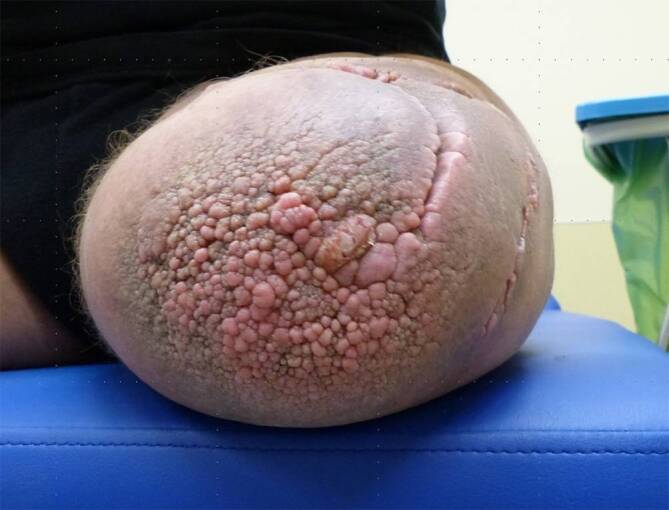
Die verruköse Hyperplasie ist eine abnorme Hautverdickung, die durch warzenartige Erhebungen und übermäßiges Wachstum des oberflächlichen Gewebes gekennzeichnet ist. Sie tritt häufig bei fehlendem Endkontakt im Stumpfendbereich auf. Diese Bereiche der Haut sind sehr vulnerabel und neigen zu Blutungen und Infektionen

- Stumpfendkontakt innerhalb des Prothesenschafts ist essenziell.
- Fehlender Endkontakt führt zu einer ungleichmäßigen Belastung der Haut, einem chronischen Weichteilödem und im Verlauf von Jahren zu einer verrukösen Hyperplasie (Abb. 5).

## Unterschenkelamputation

Als Unterschenkelamputation werden Amputationen zwischen der Syme-Amputation auf Höhe des Sprunggelenks und der proximalen Amputation in der Mitte des Ansatzes der Patellarsehne an der Tuberositas tibiae bezeichnet. Bei der ultrakurzen **transtuberösen Amputation**

transtuberösen Amputation

ist ein aktives Strecken des Kniegelenks gerade noch möglich. Die **Syme-Amputation**

Syme-Amputation

unterscheidet sich aufgrund ihrer biomechanischen Eigenschaften erheblich von einer distalen Unterschenkelamputation und zählt zu den **endbelastbaren Rückfußamputationen**

endbelastbaren Rückfußamputationen

[16].

Die Unterschenkelamputation nach Burgess ist die weltweit häufigste durchgeführte Technik. Ein myofasziokutaner langer dorsaler Unterschenkellappen, der über den knöchernen Stumpf gelegt wird, ist in den 1960er-Jahren durch Burgess als standardisierte Technik, zurückgehend auf Verduyn, beschrieben worden [17].

Die Unterschenkelamputationstechnik nach Brückner für die Patienten mit peripherer arterieller Verschlusskrankheit im Stadium IV sowie die Unterschenkelamputation nach Ertl-Dederich bei traumatischen Amputationsursache sind weitere Operationstechniken, die bei speziellen Indikationen zur Anwendung kommen [18].

### Biomechanik des Unterschenkelstumpfes

Aufgrund des wertvollen langen Hebelarms sollte immer, insofern eine suffiziente Weichteildeckung gegeben ist, ein langer Unterschenkelstumpf gewählt werden. Zudem steht bei langen Unterschenkelstümpfen eine größere Oberfläche zur Lastaufnahme im Prothesenschaft zur Verfügung, da das Stumpfende nur 20–40 % endbelastbar ist [10].

Durch die anatomischen Verhältnisse sind Amputationen im mittleren und im distalen Drittel jedoch schwierig mit Muskel, Unterhaut und Haut zu decken. Eine mechanisch belastbare Weichteildeckung des Stumpfes ist für die Prothesennutzung essenziell; somit können distale Unterschenkelamputationen, wie von Bowker et al. [18] beschrieben, durchaus problembehaftet sein und mit Wundheilungsstörungen oder **Ulzerationen**

Ulzerationen

bei der Prothesennutzung einhergehen [19]. Insbesondere bei Patienten mit höhergradiger arterieller Verschlusskrankheit und Diabetes mellitus treten im Verlauf oft Weichteilprobleme am Stumpfende auf.

Der Erhalt des eigenen **Kniegelenks**

Kniegelenks

bietet für den Patienten allerdings immense Vorteile und kann die Nachteile eines ultrakurzen Stumpfes aufwiegen. Das Kniegelenk kann vom Patienten aktiv angesteuert werden und ist einem nur durch Hüftgelenkbewegungen kontrollierbaren Prothesenkniegelenk deutlich überlegen. Zudem ist die **propriozeptive Stellungskontrolle**

propriozeptive Stellungskontrolle

des Kniegelenks im Gangzyklus von unschätzbarem Wert [20]. Allerdings kann es jedoch aufgrund des sehr kurzen Stumpfes zu Schwierigkeiten bei der Prothesenschaftanpassung und Prothesenführung kommen. Durch ein funktionelles Übergewicht der **Kniebeugemuskulatur**

Kniebeugemuskulatur

kann zudem ein Streckdefizit mit konsekutiver Kontraktur im Kniegelenk entstehen [15].

### Überblick der Unterschenkelamputationstechniken

Eine Vielzahl von transtibialen Amputationstechniken ist beschrieben und meist nach ihren Erstbeschreibern benannt. Historisch wurde der Wundverschluss durch verschiedene Muskelhautlappentechniken (Verduyn 1696; Langenbeck 1810) oder zirkuläre Schnitttechniken (Purmann 1692; Bromfield 1773) erreicht [21].

**Abb. 6:**
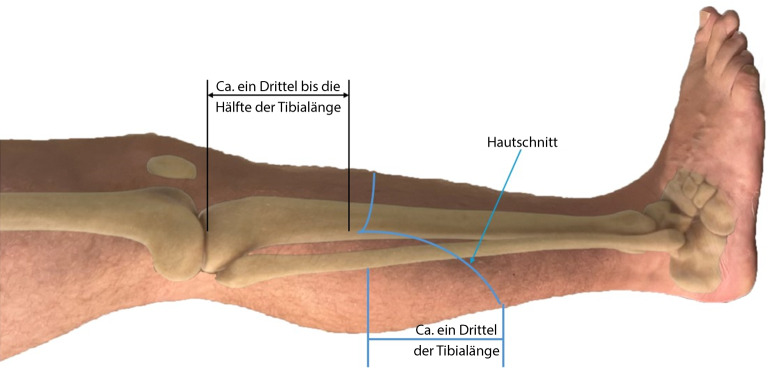
Bei der Burgess-Amputationstechnik wird ein langer myofasziokutaner dorsaler Lappen gebildet. Dieser wird über Tibia und Fibula geschlagen, und die Narbe kommt ventral zu liegen

Die Amputationstechniken nach Burgess mit Bildung eines dorsalen **myofasziokutanen Lappens**

myofasziokutanen Lappens,

einem ventralen Wundverschluss und mit Erhalt des Wadenbeins wird heutzutage in Deutschland am häufigsten durchgeführt (Abb. 6). Dabei wird die ursprünglich beschriebene Methode von Burgess ergänzt durch das zusätzliche Entfernen des M. soleus, da dieser Muskel häufig zu Venenthrombosen mit konsekutiver Perfusionsstörung neigt. Zudem atrophiert der M. soleus immer in Fettgewebe und trägt letztendlich nicht zur stabilen Muskeldeckung des Stumpfes bei [10, 17].

Untersuchungen zu subkutaner und kutaner **Durchblutung**

Durchblutung

mithilfe von Wärmebildkameras sowie die anatomische Betrachtung der Angiosome und die Beurteilung der Hautdurchblutung durch Endarterien wiesen eine häufig verminderte Perfusion der anterolateralen proximalen Unterschenkelhaut und Muskulatur nach [22, 23]. Diese Erkenntnisse führten zur Entwicklung von Amputationstechniken, wie sie in skandinavischen Ländern weit verbreitet sind. Die Amputationen nach McCollum oder häufiger Robinson beinhalten sagittale oder um 20° nach lateral **gedrehte Hautschnittführungen**

gedrehte Hautschnittführungen

im mittleren Drittel des Unterschenkels. Diese längeren Stümpfe im Vergleich zum Burgess-Stumpf sind erreichbar, da kein dorsaler langer Lappen gebildet wird, sondern der Wundverschluss analog zum Fischmaulschnitt am Oberschenkel mit kürzeren Hautlappen erfolgt. Zudem werden die tiefen Muskelschichten nicht so intensiv wie bei den dorsalen Lappenplastiken separiert, und die Wundflächen sind kleiner. Die Narben sind entsprechend am Stumpfende positioniert.

Histopathologische Untersuchungen der Unterschenkelmuskulatur durch Brückner bei Patienten mit arterieller Verschlusskrankheit im Stadium IV nach Fontaine wiesen ein häufiges Dekompensieren der anterolateralen Muskelgruppe mit konsekutiver **Nekrosebildung**

**Abb. 7:**
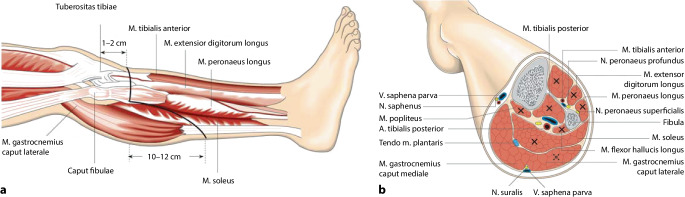
**a** Bei der Unterschenkelamputation nach Brückner erfolgt die Schnittführung analog zur Burgess-Operation, allerdings insgesamt proximaler. Da der M. gastrocnemius medialis in die Muskelloge des M. tibialis anterior gelegt wird, resultiert ein kürzerer knöcherner Stumpf. **b** Alle mithilfe eines „×“ markierten Muskeln werden bei dieser Operation entfernt. Es verbleiben nur die beiden Mm. gastrocnemii. (Aus Brückner [23])

Nekrosebildung

nach. Brückner entwickelte daraufhin eine standardisierte Unterschenkelamputation, bei der eben diese Muskelgruppe entfernt wird. Aufgrund der hierdurch fehlenden Weichteildeckung der Fibula wird diese ebenso reseziert (Abb. 7; [23]).

**Abb. 8:**
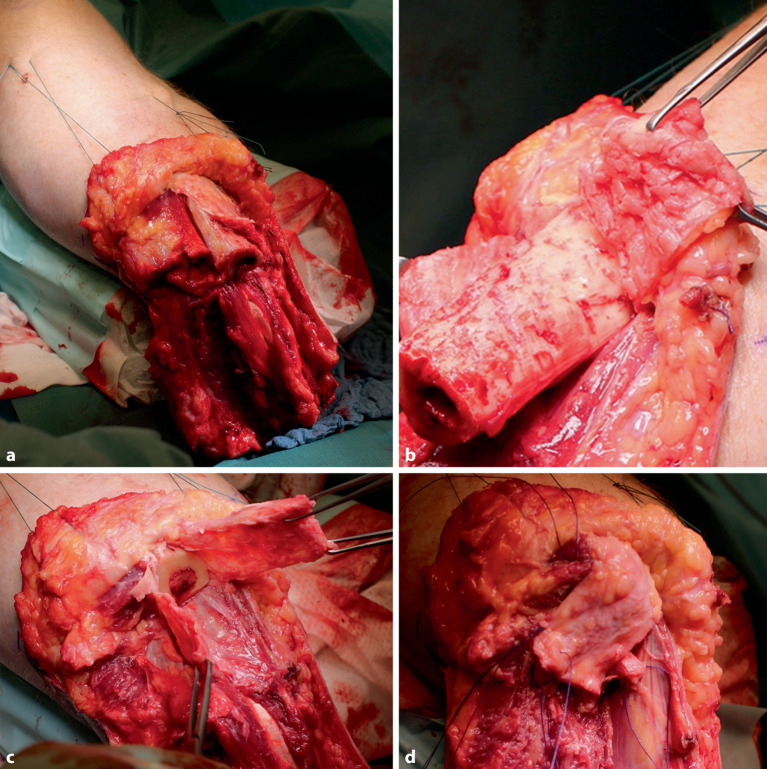
Bei der Stumpfrekonstruktion nach Ertl-Dederich wird tibiales Periost mit belassenen „Kortikalis-Chips“ genutzt, um eine schlauchförmige Periostbrücke zu etablieren. Diese wird im Sinne einer Myoplastie mit Muskulatur gedeckt. Diese Operationstechnik sollte nicht bei Patienten mit peripherer arterieller Verschlusskrankheit oder Diabetes mellitus angewendet werden. Mit dieser Technik sind Unterschenkelstümpfe bis zur Mitte des Tibiaschafts rekonstruierbar

Im Fall einer Instabilität zwischen Tibia und Fibula, wie sie bei Hochrasanztraumen auftreten kann, bewirkt die Prothesennutzung Volumenschwankungen des Stumpfes. Hier können Operationstechniken wie die nach Guedes-Pinto oder Ertl-Dederich helfen [24]. Letztere bevorzugen wir, da bei dieser Operationstechnik nur resorbierbares Fremdmaterial im Stumpf verwendet wird und kein Fremdmaterial in situ verbleibt (Abb. 8).

Zusammengefasst bieten die **posterioren Muskelhautlappentechniken**

posterioren Muskelhautlappentechniken

nach Burgess und Brückner aufgrund ihrer günstigen Narbenposition und der besseren biomechanischen Belastbarkeit Vorteile, während sagittale Schnittführungen bei komplexen Weichteilverletzungen nützlich sind. Die knöcherne Stumpfformung sollte die Belastbarkeit maximieren; bei Instabilität zwischen Tibia und Fibula sind Techniken zur **Knochenbrückenbildung**

Knochenbrückenbildung

zu erwägen.

#### Merke

- Die klassische Unterschenkelamputation in Deutschland erfolgt nach Burgess.
- Die Brückner-Technik senkt das Wundheilungsrisiko für stark durchblutungsgestörte Stümpfe.
- Bei traumatischen Amputationen nach Ertl-Dederich können lange Unterschenkelstümpfe mit hoher Belastbarkeit rekonstruiert werden.

### Stumpfvoraussetzungen zur Prothesenversorgung

Das Ziel der Unterschenkelamputation ist ein schmerzfreier, infektionsfreier und belastbarer Stumpf. Bei der Wahl der Amputationshöhe, der Planung der knöchernen Stumpfdeckung mithilfe der vorhandenen Weichteile und der Positionierung der Narben sollten folgende Stumpfkriterien angestrebt werden [15].

Haut- und Unterhaut:

- gute Durchblutung (Rekapillarisierungszeit ca. 2–3 s),
- keine Sensibilitätsstörung der Haut,
- schmerzfrei,
- Haut/Narbe ist über dem Untergrund verschieblich und reizlos,
- Lastaufnahme wird vertragen.

Weichteile:

- fixierte Muskeldeckung des Stumpfes,
- große Gefäße sind kurz proximal des knöchernen Stumpfes chirurgisch abgesetzt,
- die Unterschenkelnerven (N. tibialis, Nn. fibulares, N. suralis) sind hinreichend gekürzt und in die Weichteile verlagert.

Knöcherner Stumpf:

- die Tibiaresektionsfläche ist an den Kanten abgerundet und vorn angeschrägt,
- die Fibula ist ca. 1 cm kürzer als die Tibia und leicht von medial nach lateral ansteigend angeschrägt,
- die Membran zwischen Fibula und Tibia ist intakt.

#### Merke

**Abb. 9:**
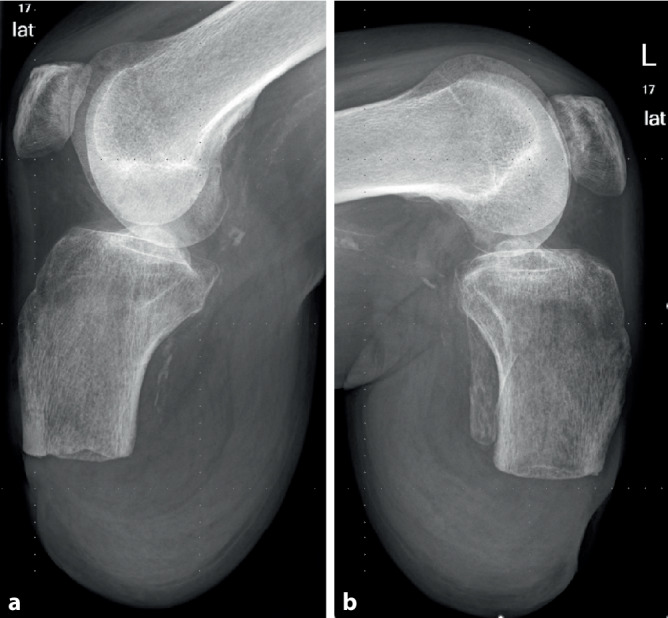
Patient mit bilateraler transtibialer Amputation. Vollständiges Fehlen des Anschrägens der Tibiavorderkante. Bei diesem Patienten wurde ca. 12 Monate aussichtslos versucht, eine prothetische Versorgung durchzuführen. Schmerzen und rezidivierende Weichteilprobleme verhinderten die Prothesennutzung

Das fehlende Abschrägen der Tibiavorderkante führt bei Prothesennutzung zu Wundheilungsstörungen und notwendiger stumpfkorrigierender Operation (Abb. 9).

Eine am Stumpfende befindliche **Narbe**

Narbe

wird bei der Lastaufnahme im Prothesenschaft immer **Distraktionskräften**

Distraktionskräften

ausgesetzt sein. Im Gegensatz hierzu wird eine ventrale Narbe automatisch komprimiert und kann besser belastet werden.

Gefäßgestielte Fasziokutanlappen, freie Muskellappen oder nichtverschiebliche Spalthautareale unmittelbar über dem knöchernen Stumpf sind langfristig mechanisch nicht gut belastbar.

Besonders wichtig ist der Bewegungsumfang im Kniegelenk. Für ein energieeffizientes und sicheres Laufen muss das Knie vollständig gestreckt werden können. Zudem sollte eine Beugung bis mindestens 80° möglich sein, um Treppen steigen und gut sitzen zu können. Ist präoperativ absehbar, dass eine gute **Kniegelenkfunktion**

Kniegelenkfunktion,

insbesondere bei ausgeprägter Beugekontraktur, nicht erreichbar ist, sollte entweder eine operative Korrektur der Kontraktur oder eine Knieexartikulation erwogen werden.

## Fazit für die Praxis

- Die Majoramputation ist kein einfaches „Abtrennen“ der Extremität, sondern eine anspruchsvolle plastisch-chirurgische Rekonstruktion.
- Die Teilhabe des Patienten nach einer Majoramputation ist unmittelbar von der Qualität des Operationsergebnisses abhängig; somit gehört eine Amputation in die Hände eines erfahrenen Operateurs.
- Um in Abhängigkeit von der Amputationsursache und des Leistungsanspruches des Patienten die optimale Versorgung zu erreichen, müssen verschiedene Oberschenkel- und Unterschenkelamputationstechniken bekannt sein.
- Eine adäquate Muskelfixierung am knöchernen Stumpfende ist nur durch die Operationstechniken Myodese und Myoplastie möglich.
- Zu viel Weichteilüberhang über dem knöchernen Stumpfende ist mit Hinblick auf eine prothetische Versorgung unbedingt zu vermeiden.

## Figures and Tables

**Abb. 10:**
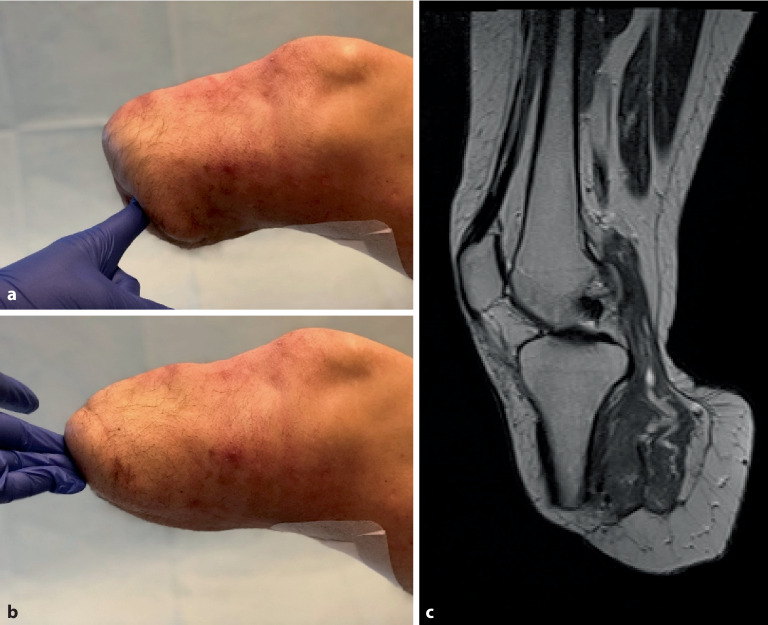
Es finden sich klinisch erheblich mobile Weichteile mit deutlicher Retrahierung hinter den Tibiastumpf (**a**, **b**). Im MRT findet sich keine fixierte Unterschenkelmuskulatur am knöchernen Stumpfende (**c**)

## CME-Fragebogen

Welche ist eine der häufigsten Ursachen für eine notwendige Majoramputation der unteren Extremität in Deutschland?

Tumorerkrankung

Traumatische Verletzung

Iatrogene Verletzung im Rahmen einer Operation

Folgen von peripherer arterieller Verschlusskrankheit

Chronische implantatassoziierte Osteomyelitis

Bei kurzen transfemoralen Majoramputationen findet sich eine typische Fehlstellung des knöchernen Stumpfes, bedingt durch die ungünstige Muskelbalance der verbliebenen Restmuskulatur. Wodurch zeichnet sich diese Fehlstellung aus?

Vermehrte Abduktion-Flexion-Außenrotation

Vermehrte Abduktion-Extension-Außenrotation

Vermehrte Adduktion-Innenrotation

Vermehrte Adduktion-Außenrotation

Vermehrte Extension-Innenrotation

Wie werden die verschiedenen Amputationsformen in Abhängigkeit vom Amputationszeitpunkt bezeichnet?

Aufgrund ausgeprägter Weichteilverletzungen oder Gefäß- und Nervenschädigungen sind trotz eines anfänglichen Erhaltungsversuches quartäre Amputationen in den ersten 4 Tagen manchmal unvermeidlich.

Erfolgt wegen der Schwere einer Verletzung die Majoramputation innerhalb von 24 h, wird dies als primäre Amputation bezeichnet.

Sekundäre Amputationen werden ab der 6. Woche nach dem Verletzungsereignis durchgeführt.

Aufgrund chronischer Infektionen oder schlechter Belastbarkeit der Extremität erfolgen auch Jahre nach dem Unfallereignis Majoramputationen, diese werden als tertiäre Amputationen bezeichnet.

Notwendige Nachamputationen aufgrund eines komplikationsbehafteten Verlaufs nach quartärer Amputation werden als quintäre Amputationen bezeichnet.

Nach welchem der folgenden Autoren ist eine Operationstechnik, wie sie bei Oberschenkelamputationen zur Anwendung kommt, benannt?

Guedes-Pinto

Ertl-Dederich

Burgess

Gottschalk

Robinson

Brückner beschreibt in seiner Unterschenkelamputationstechnik die Entfernung der kompletten anterolateralen Unterschenkelmuskulatur und der Fibula. Für welche Patientengruppe entwickelte er diese Technik?

Patienten mit einem Osteosarkom des Tibiakopfes

Patienten mit traumatischer Amputation in Höhe des Sprunggelenks

Patienten mit notwendiger beidseitiger Majoramputation

Patienten mit peripherer arterieller Verschlusskrankheit im Stadium IV

Patienten mit irreparablen Nervenschäden im Bereich des Unterschenkels

Für die Nutzung einer schaftgeführten Prothese werden ideale knöcherne Stumpfkriterien beschrieben. Welche der aufgeführten Stumpfeigenschaften sollte vermieden werden, da hieraus eine sehr schlechte Stumpfendbelastbarkeit resultiert?

Das distale Femurende ist an den Kanten abgerundet und horizontal ausgerichtet.

Das distale Femurende weist keine Osteophyten auf.

Sowohl die distale Tibia- als auch das Fibulastumpfende sind nach lateral um 45° abgeschrägt.

Die Tibiaresektionsfläche ist an den Kanten abgerundet und vorn angeschrägt.

Die Fibula ist ca. 1 cm kürzer als die Tibia und leicht von medial nach lateral ansteigend angeschrägt.

Ein 67-jähriger Patient stellt sich mit seinem Stumpf nach Syme-Amputation des rechten Beins vor. Aufgrund einer Druckulzeration, bedingt durch den Prothesenschaft, hat sich eine chronische Osteitis der frei liegenden distalen Tibia entwickelt. Als Nebendiagnosen bestehen ein diätetisch geführter Diabetes mellitus, eine geringgradige Polyneuropathie und eine arterielle Hypertonie. Welche Behandlung schlagen Sie am ehesten vor?

Eine Grenzzonenamputation unmittelbar proximal der Ulzeration, um die maximale Stumpflänge zu erreichen.

Eine distale Oberschenkelamputation ist in diesem Fall aufgrund der sichereren Wundheilungschancen zu bevorzugen.

Es sollte ein Ausheilungsversuch der Tibia-Osteitis mittels Antibiotikatherapie erfolgen.

Es sollte die proximale Unterschenkelamputation in der Technik nach Burgess erwogen werden.

Sie empfehlen eine Unterschenkelamputation nach Brückner, um einen kürzeren Stumpf zu erzielen.

Welche Aussage zur Stumpfbelastbarkeit nach Majoramputationen trifft am ehesten zu?

Unterschenkelstümpfe sind in der Regel 20–40 % endbelastbar.

Unterschenkelamputierte können sich beim Abstützen bei langen Stümpfen auch ohne Prothese voll endbelasten.

Eine Fehlstellung des knöchernen Oberschenkelstumpfes im Schaft hat keinen Einfluss auf die Endbelastbarkeit.

Die Tibiavorderkante, der knöcherne Unterrand der Patella und das Fibulaköpfchen stellen ideale lastaufnehmende Areale dar.

Die Fibula und die Tibia sollten beim Unterschenkelstumpf gleich lang sein.

Weshalb sollte bei der Unterschenkelamputation nach Burgess der M. soleus entfernt werden?

Wegen des fehlenden Ansatzes am Femur.

Um das Gefäß-Nerven-Bündel am Unterschenkel darstellen zu können.

Da der Muskel zu Venenthrombosen mit Perfusionsstörungen neigt.

Um einen sicheren spannungsfreien Wundverschluss zu ermöglichen.

Damit aufgrund der fettigen Atrophie ein längerer Stumpf resultiert.

Ein 35-jähriger Patient stellt sich 6 Monate nach traumatischer primärer Unterschenkelamputation aufgrund eines Motoradunfalls vor. Unmittelbar am Unfalltag erfolgte die Notfallamputation nach Burgess. Der Versuch einer interimsprothetischen Versorgung musste aufgrund erheblicher Schmerzen am Stumpfende und täglicher erheblicher Stumpfvolumenänderungen abgebrochen werden. Bei der klinischen Untersuchung lässt sich die Tibiaspitze unmittelbar unter der Haut tasten, und die dorsalen Stumpfweichteile sind sehr mobil. Diese können über die Stumpfspitze gebracht, aber auch mit dem Finger weit in Richtung Poplitea retrahiert werden (Abb. 10). Wie schätzen Sie die Situation unter zusätzlicher Berücksichtigung der Bildgebung ein, und welche Behandlungsempfehlung wäre am ehesten auszusprechen?

Die MRT ist unauffällig, und die Weichteile sind aufgrund einer noch nicht abgeschlossenen Stumpfkonditionierung noch nicht prothesenfähig. Es sollte eine konsequente Stumpfformung erfolgen.

Ursächlich für die Schmerzen findet sich im MRT ein deutlich erkennbares Nervus-tibialis-Stumpfneurom, das mit einer medikamentösen Schmerztherapie häufig sehr gut behandelbar ist.

Es besteht keine suffiziente Muskeldeckung des knöchernen Stumpfes. Es sollte eine plastische Stumpfrekonstruktion mit Myopexie erwogen werden.

Aufgrund der kurzen Stumpfverhältnisse und der retrahierten Muskulatur ist eine distale Oberschenkelamputation zu empfehlen.

Da die Muskulatur noch manuell über die Stumpfspitze gebracht werden kann, ist das distale knöcherne Stumpfende suffizient muskulär gedeckt. Ein konservatives Vorgehen ist zu empfehlen.
